# Supplementary material for: Genomic Selection for Live Weight in the 14th Month in Alpine Merino Sheep Combining GWAS Information
Source: Animals (Basel). 2023 Nov 14;13(22):3516. doi: 10.3390/ani13223516 (PMC10668700; doi:10.3390/ani13223516)
Supplement: Supplementary file 1 [file animals-13-03516-s001.zip › animals-2600962-supplementary.pdf]

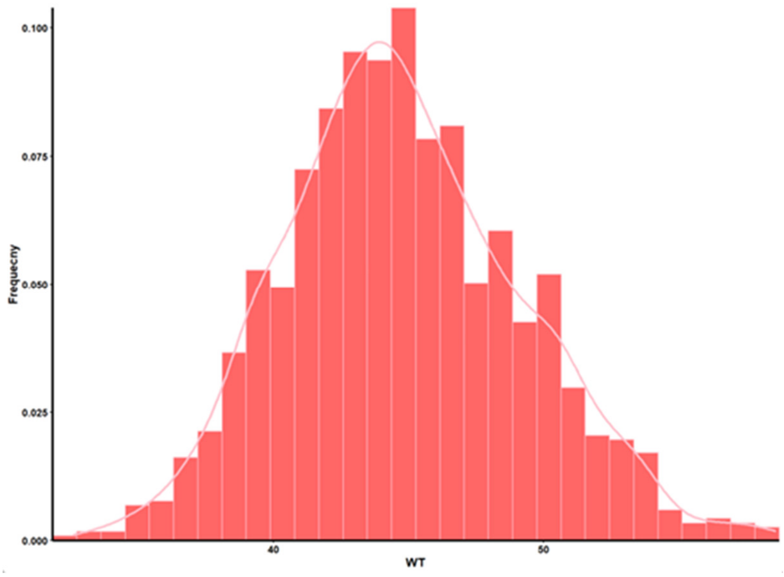

Figure S1. Histogram of weight distribution at 14 months of age, with the pink line representing the fitted normal distribution.
